# Publisher's Note

**DOI:** 10.1093/bib/bbae378

**Published:** 2024-08-05

Due to a system error, the below articles:

`A cloud-based training module for efficient *de novo* transcriptome assembly using Nextflow and Google cloud' by Ryan P Seaman, Ross Campbell, Valena Doe, Zelaikha Yosufzai and Joel H Graber (https://doi.org/10.1093/bib/bbae313)

`Reusable tutorials for using cloud-based computing environments for the analysis of bacterial gene expression data from bulk RNA sequencing' by Steven Allers, Kyle A O'Connell, Thad Carlson, David Belardo and Benjamin L King (https://doi.org/10.1093/bib/bbae301)

intended for this supplement, were published into Volume 25, issue 4 of the journal instead. The articles can be read online in the issue at https://academic.oup.com/bib/issue/25/4 and this note has been included in the supplement in place of the articles. The Publisher apologizes for the error.

